# Giant prolapsed uterine leiomyoma causing acute urinary retention and severe anemia: a case report and review of the literature

**DOI:** 10.1097/RC9.0000000000000261

**Published:** 2026-02-16

**Authors:** Yaman Almkaky, Ahmad Al-Bitar, Dema Adwan

**Affiliations:** a Faculty of Medicine, Damascus University, Damascus, Syrian Arab Republic; b Department of Obstetrics and Gynecology, Faculty of Medicine, Damascus University, Damascus, Syrian Arab Republic

**Keywords:** acute urinary retention, prolapsed fibroid, uterine leiomyoma

## Abstract

**Introduction:**

Uterine leiomyomas are common benign tumors that rarely present with severe complications. Acute urinary retention (AUR) is an exceptional initial symptom from mechanical obstruction. Profound anemia without reported menorrhagia is also atypical, resulting from a giant, prolapsed fibroid undergoing ischemic necrosis, leading to surface ulceration and chronic blood loss. We report a case of a giant prolapsed leiomyoma presenting with this rare combination of AUR and severe anemia.

**Case presentation:**

A 44-year-old, para 6, woman presented with a 1-month history of AUR. Examination revealed a large, impacted vaginal mass; labs showed severe anemia (hemoglobin 7.1 g/dL). Imaging confirmed a 13 × 12 cm prolapsed uterine leiomyoma with necrotic foci. After transfusion, a combined abdomino-vaginal myomectomy was performed. A standard vaginal approach failed, necessitating an abdominal incision with an anterior colpotomy to ligate the pedicle, followed by vaginal delivery of the mass. Histopathology confirmed a benign leiomyoma, and the patient recovered fully.

**Clinical discussion:**

This case highlights considering uterine pathology in female AUR. The patient’s retention was caused by a “cork-in-a-bottle” effect from the impacted fibroid. The severe anemia is explained by ischemic necrosis followed by surface ulceration and chronic hemorrhage from the prolapsed mass, a mechanism distinct from menorrhagia. The surgical management demonstrates the necessity of intraoperative adaptability. The innovative use of an anterior colpotomy via an abdominal approach provided safe access to the vascular pedicle after a standard vaginal approach failed, bypassing the distorted anatomy.

**Conclusion:**

This case underscores that giant leiomyomas should be in the differential for female AUR, especially with concurrent anemia. It clarifies the pathophysiology of severe anemia without menorrhagia from a prolapsed, necrotic fibroid. Finally, it champions surgical flexibility, showing how an innovative, combined abdomino-vaginal approach can overcome significant anatomical challenges to ensure a successful outcome.

## Introduction

Uterine leiomyomas, colloquially known as fibroids, are the most prevalent benign solid tumors of the female genital tract. Their clinical and public health significance is underscored by an exceptionally high incidence, particularly during the reproductive and perimenopausal years. Landmark studies utilizing ultrasound screening have revealed a cumulative incidence by age 50 of nearly 70% in white women and exceeding 80% in women of African ancestry. This high prevalence establishes leiomyomas as a condition that a majority of women will experience in their lifetime^[^1–3^]^.

HIGHLIGHTS

- Uterine leiomyomas should be a differential diagnosis for acute urinary retention in women.
- Severe anemia can result from ischemic necrosis and surface ulceration of the fibroid, even without patient-reported menorrhagia.
- Surgical flexibility is crucial. A novel combined abdomino-vaginal approach with an anterior colpotomy proved effective for this large, impacted mass and represents a valuable technique for complex cases.

Recent global health data further highlight the growing burden of this condition. Analysis from the Global Burden of Disease Study (1990–2019) indicates a steady increase in the global age-standardized incidence rate of uterine fibroids, with an estimated average annual percent change of 0.25. This trend suggests that the impact of leiomyomas on women’s health and healthcare systems worldwide is expanding. The epidemiology of leiomyomas is marked by significant racial and ethnic disparities. The risk is consistently reported to be two to three times higher in Black women compared to white women. This disparity extends beyond mere incidence; Black women tend to be diagnosed at a younger age, present with a greater tumor burden, and are more likely to experience severe symptoms, including profound blood-loss anemia, necessitating more frequent and often more invasive interventions^[^2–5^]^.

Leiomyomas are monoclonal tumors originating from a single smooth muscle cell (myocyte) within the myometrium. Their growth is fundamentally driven by the female sex hormones, estrogen and progesterone, which explains their proliferation during the reproductive years and their typical regression after menopause^[^6^]^.

The clinical presentation of leiomyomas is highly variable and is largely dictated by their number, size, and location within the uterus. While the majority of women with fibroids remain asymptomatic, an estimated 25–30% experience debilitating symptoms that significantly impair their quality of life and necessitate medical or surgical intervention. When symptoms do manifest, they most frequently include abnormal uterine bleeding (AUB) – typically presenting as heavy or prolonged menstrual bleeding (menorrhagia) – and bulk-related symptoms. These bulk symptoms arise from the mass effect of the tumor on adjacent pelvic organs and can manifest as pelvic pain, a sensation of pressure or fullness, constipation, and chronic lower urinary tract symptoms such as frequency and urgency. This common clinical picture establishes a baseline of expectation for clinicians managing these tumors^[^6–11^]^.

While chronic urinary symptoms are a recognized consequence of large uterine fibroids, the sudden and complete inability to void, known as acute urinary retention (AUR), is a rare and dramatic initial presentation. The medical literature documents this phenomenon primarily through individual case reports and small case series, with 1 review identifying only 37 reported cases of uterine leiomyomas causing AUR, confirming its status as an exceptional event in gynecological practice^[^11^]^.

Equally atypical is the development of severe anemia in the absence of a clear patient history of menorrhagia^[^8^]^.

Here we present a 44-year-old woman presenting with AUR and severe anemia due to a giant, impacted uterine leiomyoma, who was successfully treated with a combined abdomino-vaginal myomectomy.

This case report has been reported in line with the SCARE checklist^[^9^]^.

## Case presentation

A 44-year-old Arab woman, who has had six vaginal deliveries, presented to our department complaining of urinary retention for 1 month, which was managed with intermittent urinary catheterization as needed. She also reported discomfort in her lower abdomen. The patient had no significant past medical, drug, or surgical history.

Upon clinical examination, her abdomen was soft and non-tender with no other pathological findings. A vaginal examination revealed a solid mass obstructing the vaginal opening. The patient’s vital signs were stable: blood pressure was 120/80 mmHg, pulse was 86 bpm, temperature was 37°C, and oxygen saturation was 98%.

An abdominal and pelvic ultrasound was performed, which showed a lesion on the posterior wall of the uterus (within the myometrium). The lesion had a heterogeneous echo pattern with internal calcifications and showed vascularity. It measured approximately 12 × 9 × 13 cm, with a volume of about 520 cm^3^. The right and left adnexa were normal, with no cystic or solid masses. There was no free fluid in the abdomen.

Blood tests were drawn and showed the following results: hemoglobin 7.1 g/dL, hematocrit 25.1%, platelets 212 × 10^3^µL, creatinine 0.7 mg/dL, and urea 17 mg/dL. The remaining lab values were within the normal range.

**Figure 1.**
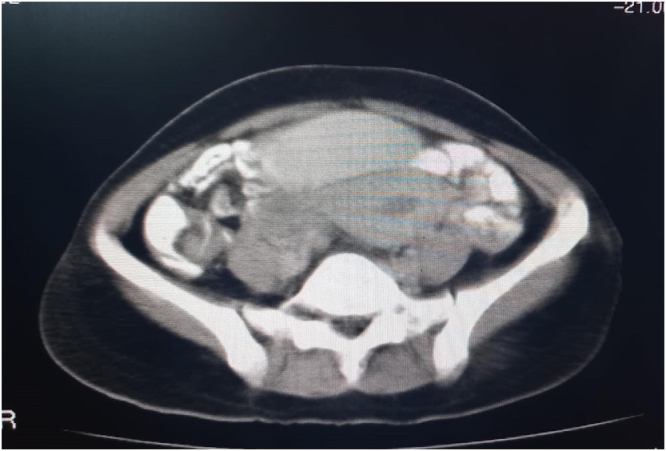
CT Scan of the pelvis showing a large, heterogeneous soft-tissue mass.

The patient was prepared for a CT scan of the chest, abdomen, and pelvis with contrast. The scan revealed a soft tissue mass with heterogeneous density and several necrotic foci, measuring 9 × 12 × 13 cm (Fig. 1A-D). The mass was displacing the bladder anteriorly and superiorly and was most consistent with a fibroid tumor. The rest of the findings were normal with no other signs of disease. While imaging suggested a posterior uterine wall origin, the severe anterior displacement of the bladder and the mass’s descent into the vagina indicated a broad-based pedicle likely originating from the lower uterine segment or cervix.

The patient was admitted to the hospital for surgical preparation. She received two units of blood and one unit of plasma, which improved her hemoglobin to 11.2 g/dL, hematocrit to 34.3%, and platelets to 215 × 10^3^µL.

The patient underwent surgery with spinal anesthesia. An attempt was made to deliver the fibroid vaginally by rotating it, but this proved difficult due to the large size of the tumor and its impaction within the vaginal canal, making it impossible to reach the tumor’s pedicle.

**Figure 2.**
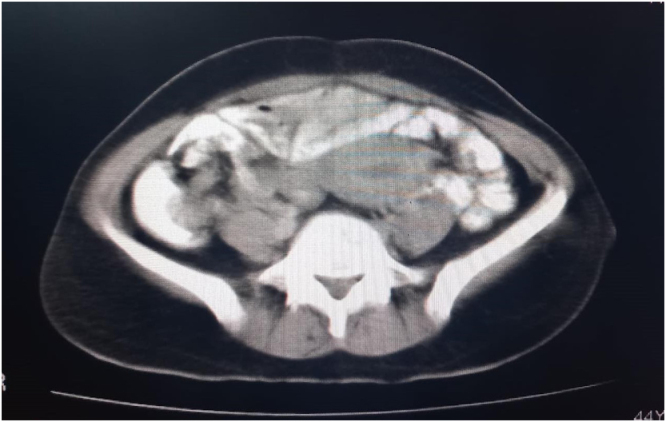
CT Scan of the pelvis showing a large, heterogeneous soft-tissue mass.

The decision was made to convert to a laparotomy (Fig. 2A-B). A Pfannenstiel incision was made, and the peritoneal cavity was entered. The uterus was identified, and the bladder was carefully dissected off the lower uterine segment and cervix, which were pulled high into the abdomen by the prolapsed mass. To safely access the fibroid’s pedicle, a 3 cm longitudinal incision was made in the anterior vaginal wall (anterior colpotomy), just below the cervico-vesical reflection. This maneuver provided direct visualization of the fibroid’s broad, approximately 2-cm wide pedicle, which was attached to the posterior lip of the cervix. The pedicle was isolated and ligated using a combination of suture ligation and bipolar coagulation. After securing hemostasis, the devascularized mass was gently pushed from above and successfully delivered through the vaginal introitus. The anterior colpotomy was closed in layers with absorbable suture. A zigzag drain was placed in the pouch of Douglas, the abdominal cavity was irrigated, and the abdomen was closed in layers.

**Figure 3.**
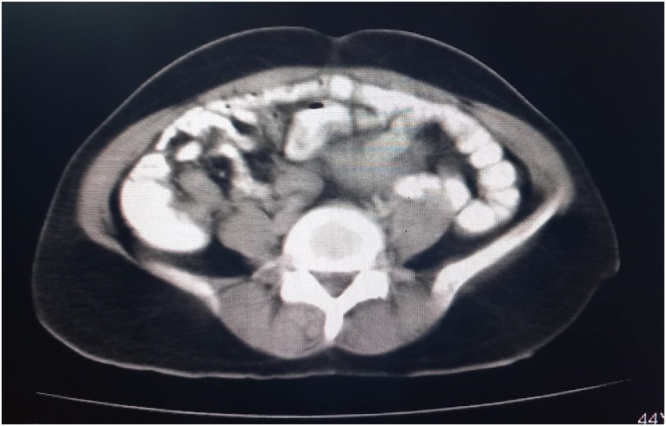
CT Scan of the pelvis showing a large, heterogeneous soft-tissue mass.

The genital tract was inspected, and existing lacerations were repaired; an external abrasion on the midline was also sutured.

**Figure 4.**
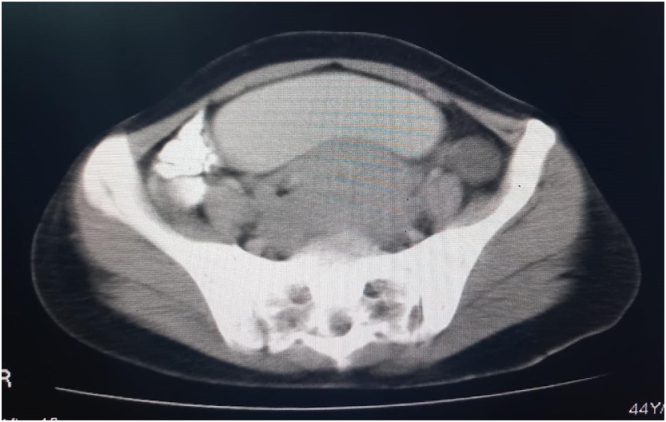
CT Scan of the pelvis showing a large, heterogeneous soft-tissue mass.

The specimen was sent to the pathology department, and the histopathological examination confirmed that the tumor was a benign leiomyoma, 13 × 12 cm, with no malignancy (Fig. 4A,B).

**Figure 5.**
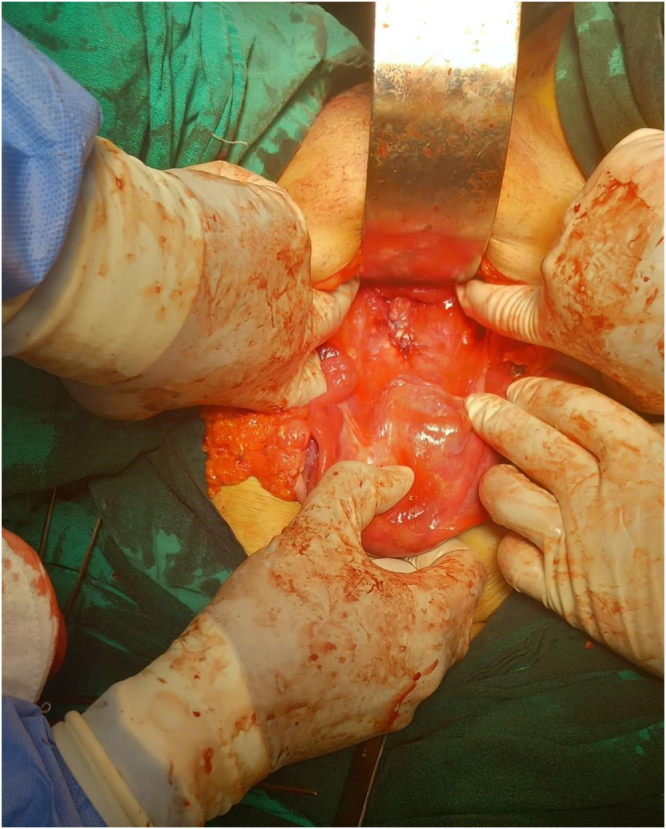
Intraoperative view during laparotomy.

The patient was discharged the next day in good general condition with a hemoglobin level of 10 g/dL.

**Figure 6.**
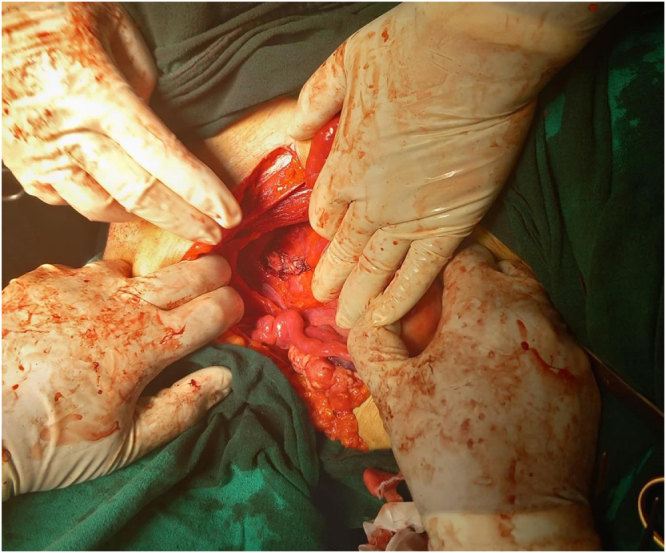
Intraoperative view during laparotomy.

**Figure 7.**
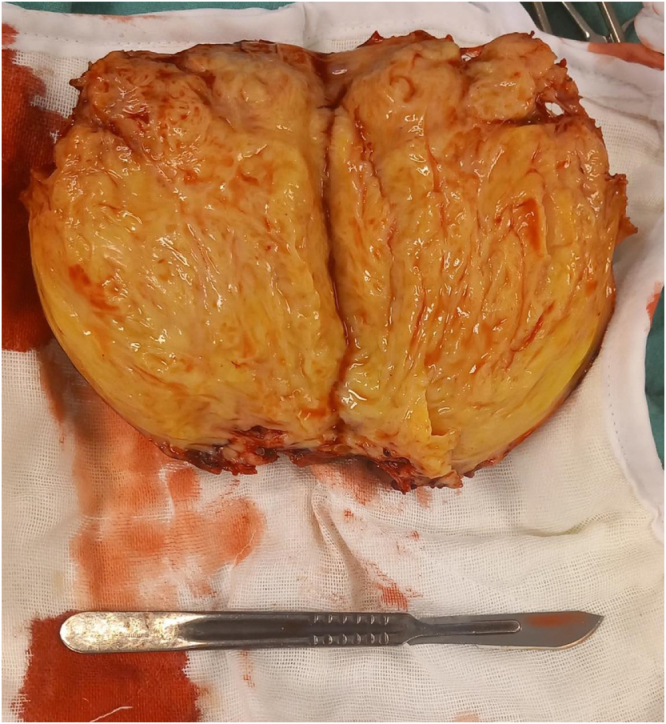
Gross specimen.

**Figure 8.**
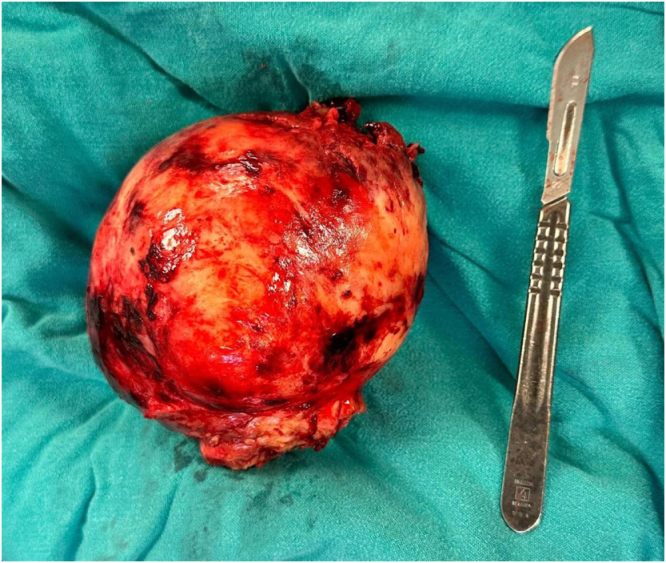
Gross specimen.

The patient presented in good health for their 1-month follow-up and will continue to be monitored regularly.

## Discussion

This case illustrates a rare but important clinical scenario where a common benign pathology, uterine leiomyoma, presents with an uncommon and severe combination of complications: AUR and profound anemia. The successful management hinged on meticulous preoperative evaluation, correction of a significant systemic derangement, and, most critically, a high degree of intraoperative adaptability to overcome unforeseen surgical challenges.

AUR is an exceptional presenting symptom for uterine fibroids. While a significant proportion of women with large fibroids report chronic lower urinary tract symptoms like frequency and urgency, the complete inability to void is far less common. The incidence of AUR as the primary complaint leading to the diagnosis of a fibroid is low enough that it is primarily documented through individual case reports and small case series in the medical literature. This case is therefore valuable as it contributes to the body of evidence on this rare presentation and underscores the importance of including uterine pathology in the differential diagnosis of AUR in women^[^10–15^]^.

The differential diagnosis for a large pelvic mass in a perimenopausal woman causing obstructive uropathy is broad and must include both benign and malignant etiologies. Beyond the more common considerations of ovarian neoplasms and uterine sarcomas, clinicians must also consider rarer entities. For instance, primary leiomyoma of the urinary bladder, though very uncommon, can present with identical symptoms of AUR. Therefore, a systematic diagnostic approach is essential^[^15–19^]^.

In this context, advanced cross-sectional imaging plays a pivotal role. While pelvic ultrasound provided the initial characterization of the mass as uterine in origin, the contrast-enhanced CT scan was invaluable for several reasons. First, it precisely defined the tumor’s dimensions and its anatomical relationship to adjacent structures, particularly the severe anterior displacement of the bladder and the potential for ureteric compression. Second, it provided crucial information about the tumor’s internal characteristics. The identification of internal necrotic foci, while raising a low suspicion for malignancy, also offered a plausible explanation for the patient’s severe anemia in the absence of reported menorrhagia. This detailed preoperative anatomical and pathological roadmap was crucial for anticipating the surgical complexity and for formulating a flexible and safe surgical plan^[^19–24^]^.

### Pathophysiological insights: a dual mechanism of morbidity

The literature describes two principal mechanisms by which fibroids can cause AUR. The first involves posterior or fundal fibroids that cause the entire uterus to become acutely retroverted and incarcerated within the sacral hollow, which in turn elongates and kinks the urethra. The second, more direct mechanism, involves a cervical or lower uterine segment fibroid that grows to a sufficient size to cause direct compression of the bladder neck and urethra against the posterior aspect of the pubic symphysis. Less common theories have also been proposed, including pelvic congestion related to hormonal changes, detrusor muscle ischemia due to a “vascular steal” effect by the highly vascular tumor, and stretching of the pudendal and sacral nerves that innervate the bladder^[^22–26^]^.

The current case represents a severe and complex example of the direct compression mechanism. The fibroid’s giant size and its origin from the low posterior uterine wall created a “cork-in-a-bottle” effect. It was simultaneously impacted within the bony pelvis, causing direct urethral compression, and prolapsed into the vagina, obstructing any attempt at manual reduction or simple surgical access from below. This dual impaction and prolapse created a formidable mechanical obstruction that was unlikely to resolve without definitive surgical removal^[^21^]^.

One of the most instructive aspects of this case is the severe anemia (hemoglobin 7.1 g/dL) in a patient who did not report a history of menorrhagia. This apparent contradiction highlights a crucial pathophysiological process distinct from typical AUB. A clear, multistep cascade can be constructed by synthesizing evidence from the literature on fibroid biology and the complications of prolapse.

The process begins with vascular insufficiency. Giant leiomyomas possess an aberrant and often tenuous blood supply that can become inadequate to support the tumor’s large mass. As the tumor outgrows its vascularity, it undergoes ischemic degeneration and central necrosis. This intrinsic pathological process was confirmed in our patient by the CT scan, which revealed multiple necrotic foci within the tumor.

This process is then critically compounded by the fibroid’s prolapse. A submucosal or cervical fibroid that protrudes through the cervix is no longer in the sterile, protected environment of the uterine cavity. It is exposed to the vaginal microbiome and to constant mechanical friction and pressure. The necrotic, devitalized surface of such a prolapsed mass is exceptionally vulnerable to pressure necrosis, surface ulceration, and secondary infection. This creates a large, raw, friable, and chronically inflamed surface^[^19,20^]^.

This ulcerated surface becomes a source of chronic, low-grade, noncyclical hemorrhage. This is not menstrual bleeding; rather, it is a constant, slow ooze of blood from the tumor’s surface directly into the vagina. This type of occult blood loss can be substantial over weeks or months, leading to profound iron-deficiency anemia. The patient may not perceive this slow, continuous loss as a “heavy period,” which explains the discrepancy between the severe anemia and the reported menstrual history. This pathophysiological sequence – ischemic necrosis compounded by prolapse-induced ulceration leading to chronic surface hemorrhage – provides a comprehensive explanation for the clinical triad observed in this patient: a giant fibroid with necrotic changes, severe anemia, and a self-reported absence of menorrhagia^[^26–29^]^. This represents a key teaching point for clinicians evaluating patients with similar presentations.

The surgical management of giant, impacted pelvic masses requires not only a sound preoperative plan but also the willingness and ability to adapt that plan based on intraoperative findings. The surgical narrative of this case serves as a powerful lesson in flexible, problem-solving surgery^[^17^]^.

The management of complex myomas relies on the surgeon’s ability to select and combine techniques from a “surgical toolbox” as the situation dictates. The initial decision to attempt a vaginal myomectomy was logical and represents the standard, least invasive approach for a prolapsed pedunculated fibroid. This approach is well-described, including in a 2005 report by Guzin *et al* detailing the vaginal removal of a huge prolapsed pedunculated submucous leiomyoma^[^30^]^. However, this standard tool is often inadequate for giant, impacted, or broad-based fibroids due to the high risk of incomplete removal, uncontrollable hemorrhage from the stalk, or iatrogenic uterine inversion from excessive traction. The immediate failure of the vaginal approach in this case confirmed the severity of the impaction and correctly stratified the case as one requiring a more advanced strategy^[^12^]^.

The conversion to an abdominal approach provided superior access and control. However, a standard abdominal myomectomy or hysterectomy would have been fraught with peril. The tumor’s extremely low position and the severe anatomical distortion, with the bladder pulled high into the abdomen, would have placed the bladder and ureters at an exceptionally high risk of injury during dissection. It is in this context that the surgical team’s innovative solution becomes apparent. The literature describes several advanced techniques for such challenging cases, including laparotomy with a posterior colpotomy to preserve the uterus in a nulliparous woman, a preliminary isthmic incision to access the stalk and facilitate a safer hysterectomy, or even pre-emptive uterine artery ligation to minimize blood loss with extremely large tumors^[^11^]^.

The technique employed in this case – an abdominal approach to create an anterior colpotomy – is another elegant and effective tool in this advanced surgical toolbox. This technique differs from the purely vaginal approach used in the 2005 case by Guzin *et al*, as it was necessitated by the failure of vaginal access due to impaction^[^30^]^. This maneuver ingeniously bypassed the impacted portion of the tumor and the displaced bladder, providing direct, safe, and controlled access to the vascular pedicle. Once the pedicle was ligated and the tumor was devascularized, the final step of delivering the mass through the vagina was both logical and safe. It minimized the need for a large abdominal incision for specimen extraction and avoided excessive traction on the pelvic organs and their vascular supply^[^22,31–34^]^. This case serves as a compelling argument against rigid adherence to a single surgical plan, championing instead the surgeon’s readiness to be flexible and to combine techniques from different approaches (abdomino-vaginal) to achieve the safest possible outcome.

Table 1 highlights that while various surgical techniques are employed for giant fibroids causing AUR, the unique combination of giant size, prolapse, severe impaction, and profound anemia in the current case necessitated a novel, combined surgical solution not commonly detailed in other reports.

**Table 1:** Review of giant uterine leiomyoma presenting with acute urinary retention

Author (Year)	Patient age(years)	Parity	Tumor size & location	Key clinical features	Surgical approach	Outcome/Key learning
Ibeanu *et al* (2009)^[^1^]^	41	1	20 cm, posterior fundal	AUR	Total abdominal hysterectomy (TAH)	Resolution of symptoms
Mirza *et al* (2020)^[^2^]^	45	3	20 × 15 cm, cervical	AUR	TAH	Resolution of symptoms
Shrestha *et al* (2017)^[^3^]^	40	2	15 × 12 cm, posterior wall	AUR	Abdominal myomectomy	Resolution of symptoms
Singh *et al* (2016)^[^4^]^ – Case 1	42	3	7.8 × 6 cm, posterior subserous	AUR, bilateral hydronephrosis	TAH with bilateral salpingo-oophorectomy	Hydronephrosis resolved post-op
Singh *et al* (2016)^[^4^]^ – Case 3	25	0	15 × 10 cm, cervical	AUR, bilateral hydroureteronephrosis	Abdominal myomectomy	Hydronephrosis resolved post-op
Guzin *et al* (2005)^[^30^]^	NR	NR	Huge, prolapsed, pedunculated submucous	NR	Prolapsed fibroid	Vaginal myomectomy
Current case	44	6	13 × 12 cm, posterior, prolapsed	AUR, severe anemia (Hb 7.1), impaction	Combined abdomino-vaginal myomectomy via anterior colpotomy	Resolution of symptoms. Highlights surgical adaptability

## Conclusion

This report details the successful management of a rare presentation: AUR and severe anemia caused by a giant, impacted uterine leiomyoma. The case provides critical lessons for clinicians, clarifying that severe anemia in such patients can arise from occult blood loss due to ischemic necrosis, a key finding when menorrhagia is absent. Most importantly, it highlights the value of surgical adaptability. The successful outcome required an unconventional combined abdomino-vaginal strategy, demonstrating an innovative approach for managing complex, impacted pelvic masses.
